# Infants without health insurance: Racial/ethnic and rural/urban disparities in infant households’ insurance coverage

**DOI:** 10.1371/journal.pone.0222387

**Published:** 2020-01-24

**Authors:** Scott R. Sanders, Michael R. Cope, Paige N. Park, Wesley Jeffery, Jorden E. Jackson

**Affiliations:** Department of Sociology, Brigham Young University, Provo, Utah, United States of America; Johns Hopkins School of Public Health, UNITED STATES

## Abstract

In order to gain insights into how the effects of the uneven adoption of Medicaid expansion varies across the rural/urban spectrum and between racial/ethnic groups in the United States, this research used the fertility question in the 2011–2015 American Community Survey to link infants’ records to their mothers’ household health insurance status. This preliminary exploration of the Medicaid expansion used logistic regression to examine the probability that an infant will be born without health insurance coverage. Overall, the states that adopted Medicaid expansion improved the health insurance coverage for households with infants. However, rural households with infants report lower percentages of coverage than urban households with infants. Furthermore, the rural/urban gap in health insurance coverage is wider in states that adopted the Medicaid expansion. Additionally, Hispanic infants remain significantly less likely to have health insurance coverage compared to Non-Hispanic White infants. Understanding infant health insurance coverage across ethnic/racial groups and the rural/urban spectrum will become increasingly important as the U.S. population transitions to a minority-majority and also becomes more urban. Although not a perfect solution, our findings showed that the Medicaid expansion of health insurance coverage had a mainly overall positive effect on the percentage of U.S. households with infants who have health insurance coverage.

## Introduction

In this paper is a preliminary exploration of how the uneven expansion of Medicaid affects racial/ethnic health insurance coverage across the rural/urban spectrum in the United States. However, unlike previous studies that have examined health insurance coverage in the United States [1, 2, 3], we emphasized only those households with infants. It is essential to understand the settings in which infants are born because previous research has shown that access to Medicaid improves health outcomes [4] and their household environment has long-term implications for their educational achievement, positive developmental trajectories, and transitions to productive adult roles [5, 6]. The absence of basic and essential healthcare *in utero* or during early formative stages can also create barriers that adversely affect health and cognitive development [7]. Furthermore, many households with infants qualify for health insurance coverage, but they fail to obtain it due to language barriers or being unfamiliar with the process required to get insurance [8].

Understanding how health insurance in households with infants varies across the rural and urban spectrum, as well as between ethnic/racial groups, can help to identify which infants might face social or geographic healthcare barriers. Therefore, understanding the prevalence, ethnic/racial, and spatial distribution of infants born to mothers without health insurance can provide insights into future demographic, labor, and public health issues in the United States, while also building on the current understanding of infant health insurance [9].

Within this context, our paper had three specific objectives: 1) to highlight spatial patterns of health insurance for infants across the rural/urban spectrum; 2) to produce an empirical baseline across ethnic/racial variation in health insurance among America’s infants; 3) to estimate the effects on health insurance of residing in a state that expanded Medicaid coverage.

## The medicaid expansion and barriers to health insurance coverage

Disparities between rural and urban health insurance have long been a concern. Before Medicaid, rural residents were more likely to be uninsured than were urban residents [10, 11] and one of the policy goals associated with the Medicaid expansion was to decrease this disparity in coverage. Rural communities frequently include higher proportions of low-to-moderate-income individuals and employ more workers in jobs less likely to provide employer-sponsored insurance than do urban communities [12]. As such, rural residents stood to benefit significantly from the Medicaid expansion. Also, because of the long-standing disadvantages in access to care, coverage, and generally poorer health status among rural populations, the US Government-funded outreach programs that specifically targeted rural enrollment [13, 14].

However, despite efforts in improving rural access to care, increases in the percentage of insured rural households, urban populations continue to reflect a higher percentage of insured households overall [15]. Furthermore, the continued macro urban/rural coverage disparity is attributable in part to the lack of the Medicaid expansion in predominantly rural states. Although rural populations have higher proportions of adults who can benefit from Medicaid expansion, they are less likely than are urban populations to live in states that have expanded Medicaid coverage [12]. Only 38% of rural, low-income adults live in Medicaid expansion states, while 50% of urban low-income adults live in these states. As a result, compared to urban residents, rural residents are less likely to benefit overall from the healthcare expansions provided by the Medicaid expansion [11].

Racial and ethnic disparities in coverage still exist, and the lack of health insurance is a primary factor in Hispanics’ and Blacks’ decreased likelihood to sign up for healthcare compared to Non-Hispanic Whites [16, 17]. Before the Medicaid expansion, Non-Hispanic Whites were more likely to be insured than Blacks and Hispanics. After the Medicaid expansion, uninsured levels fell for all racial groups, but by higher percentages for ethnic minority groups than for Non-Hispanic White populations. Despite this reduction in the disparity gap among these populations, Non-Hispanic Whites are still more likely to be insured than Blacks or Hispanics [2, 18]. Furthermore, Monnat [19] found that rural Hispanic populations have lower percentages of health insurance than do urban Hispanic populations. Thus, despite the expansion of healthcare, disparities in health insurance coverage persist.

Other research examined how health insurance coverage varies between rural/urban and racial/ethnic groups, but these studies do not provide insight into the variance in health insurance for households with infants. Thus, this research sought to address this gap in the health insurance and infant well-being literature.

## American community survey: Fertility and Health insurance data

In this paper, we preliminarily explore how the Medicaid expansion affected households with infants by utilizing the fertility question in the 2011–2015 ACS to link infants’ records to their mothers’ health insurance status. The specific data used in the analysis were the 2011–2015 five-year data downloaded from the Integrated Public Use Microdata Series (IPUMS) U.S. database [20]. The 2011–2015 ACS is the most recent five-year sample, and when compared to one-year ACS samples, the data allows for a more robust analysis across rural/urban areas and racial/ethnic groups.

Using this dataset is particularly advantageous because IPUMS harmonizes U.S. Census Bureau data in order to improve the ACS five-year sample’s consistency and accuracy. A limitation of our data is that the Medicaid expansion under the ACA occurred in 2014. Therefore, part of the data sample only captures the first two years of this expansion. However, using the most recent 2011–2015 ACS microdata files allowed this research to provide a baseline effect of the Medicaid expansion for rural/urban areas and racial/ethnic groups. Establishing a baseline estimate of the Medicaid expansion is especially important, given the ongoing changes to the U.S. healthcare system.

Turning to infant health insurance coverage, we used the ACS five-year estimates for 2011–2015 microdata files in order to identify infants and their mothers. The ACS five-year estimates for 2011–2015 have a sample size of 15,637,457. To subset the file to only households with infants, we first defined infants as aged one year or younger at the time of the survey. Second, we linked these infants with their mothers’ and households’ information by merging their files with the other two files. Third, in order to ensure that infants were properly linked to their biological mothers, we used the ACS fertility question (i.e., “Has this person given birth to any children in the past 12 months?”) together with ACS individual and household ID variables. This research builds on previous work that established this method with which to examine the household conditions of infants and infant wellbeing [21, 22]. The process subsetted the full ACS five-year estimates for 2011–2015 to the final sample of 179,251 infants that we used in our analysis.

Once we identified households with infants, we used the ACS health insurance questions to determine whether or not an infant was born to a mother without health insurance. Beginning in 2010, the ACS asked respondents if they had health insurance. For this study, insurance coverage was based on the IPUMS “Any health insurance coverage” variable that indicates whether or not the respondent and household had any health insurance coverage at the time of the interview, as measured by employer-provided insurance, insurance purchased privately, Medicare, Medicaid or other governmental insurance, TRICARE, Veterans Administration-provided insurance, or other military health insurance.

In constructing the health insurance coverage variable, the U.S. Census Bureau does not consider respondents to have coverage if their only coverage derives from Indian Health Services (IHS), because IHS policies are not always comprehensive. The ACS data used to construct the health insurance coverage variable, and other variables, comes from IPUMS. IPUMS cleans and edits the healthcare insurance variable further in order to improve the responses’ accuracy. For example, IPUMS examines responses to questions about military service and Medicaid eligibility, and edits healthcare insurance based on family and household relations. For a more detailed description of the household health insurance status variables, see IPUMS, 2019, and Lynch, Boudreaux, and Davern, 2009. Finally, even in the unlikely case that all Native American infants have IHS and receive care through it, because the number of Native American infants is a relatively small percentage of the total sample, not coding IHS as insurance would have little effect on the overall findings.

## Measurements

**Fig 1:**
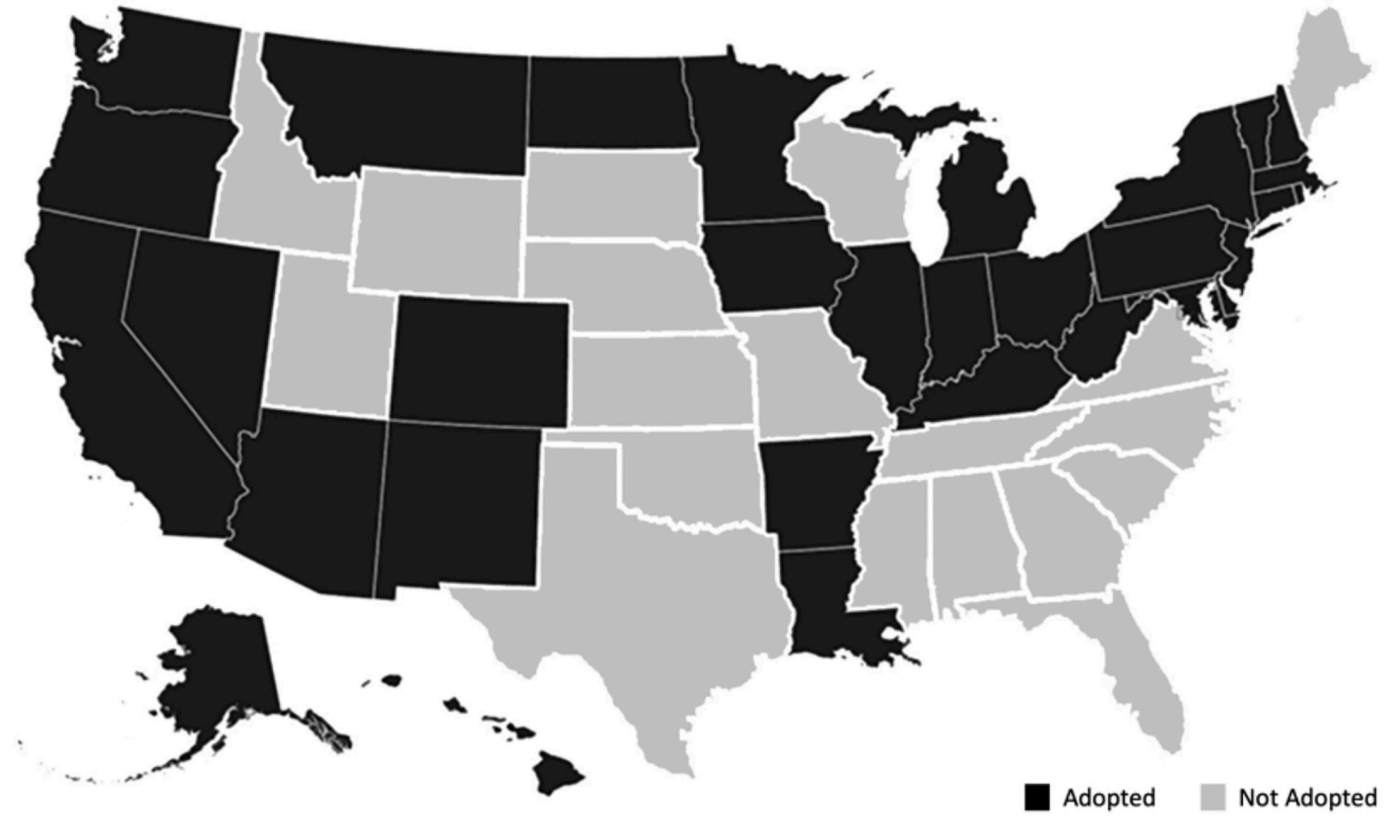
State status of medicaid expansion, 2015.

The *rural/urban* variable was based on the ACS metro variable that used the Office of Management and Budget’s definition of an area with a population greater than 50,000 as a metropolitan area and principal city. The ACS determines whether a household is in a metropolitan area by examining the Public Use Microdata Area (PUMA). The *rural/urban* variable used in the analysis coded for all households defined in the ACS as “Not in a metro area” and as “1” (rural), while all other households were coded “0” (urban). The *resides in a state without the Medicaid expansion* variable was based on whether or not a state had opted to expand Medicaid coverage by 2016 [23, Fig 1]. *Race/ethnicity* data were based on ACS demographic categories. While race/ethnicity does not define the identity of an individual, it does represent a shared seriality or level of social life and shared historical experience with social structures [24]. Therefore, using this understanding of seriality, we used the ACS race and ethnicity categories to facilitate a macro-level analysis. The race and ethnicity variables were coded by identifying all Hispanic infants from the ACS’s question regarding Hispanic origin. Self-identifying Hispanic individuals can be of any race. All other infants were classified as Non-Hispanic (i.e., Non-Hispanic Whites, Non-Hispanic Blacks, etc.). In order to better understand variances in health insurance, a variety of demographic variables were added, including the mother’s self-reported age, immigration situation, and marital status. The number of siblings in the household at the time of the survey was also included to account for households’ composition. Also, we considered several factors commonly associated with maternal human capital and employment.

Specifically, dummy variables indicated whether or not infants’ mothers self-reported their ability to speak English and coded as “1” (with speaks English well, very well, or only when combined as the reference category and code as “0”); whether or not the mothers self-reported having a high school education or less, or alternatively having some high school, coded as “1” (with college graduates serving as the reference category coded “0”); and whether or not their mothers were employed. We expected that higher levels of human capital and maternal employment would decrease the likelihood that an infant would live in a household without health insurance. Finally, we also controlled for total household income per USD 1,000.

## Analytical framework

First, we identified the percentage of infants in households without health insurance in rural and urban areas, as well as for each race/ethnicity, and then we showed how these percentages varied, and whether or not the infants lived in a state that had expanded health insurance via the Medicaid expansion.

Next, we generated a multivariate logistic regression that estimated the likelihood of not having health insurance and controlled for maternal demographics and other household characteristics. The logistic regression was appropriate for our purposes because the probability of an infant being born to a mother with or without health insurance is dichotomous [25].

Finally, we ran a logistic regression with an interaction between rural/urban Hispanic ethnicity, and rural/urban Medicaid expansion variables to more fully understand the ethnic-geospatial disparities in infant health insurance in the United States. The predicted probabilities of each interaction were then graphed for improved interpretation because even though a significant interaction variable in a logistic regression model indicates that the effect of a variable is not the same for all values of the interacting variable, the reported values do not provide clear information about the nature of the interaction. Graphing the interaction provides a more complete understanding of the nature of the interaction [26].

Similar to previous research that has used the ACS fertility question in order to link infants to their mothers’ socioeconomic characteristics, logistic regressions adjust for the design effects of the ACS using the ACS-generated, personal-level replicate weights, which ensures that the results are consistent with the population estimates from the U.S. Census Bureau’s Population Estimates Program [22, 27].

## Results

Table 1 describes infants born into households without health insurance in rural/urban areas according to race/ethnicity and whether or not their home states had implemented the Medicaid expansion. The overall results indicated that approximately 17.4% (572,277 weighted households) of all infants are born into households with no form of health insurance. A nearly significant (*p* < .06) discrepancy emerged between rural and urban infants, in which approximately 19.9% (70,224 weighted households) of rural infant households had no health insurance compared to 16.8% (502,053 weighted households) of urban infant households.

**Table 1:** Percentage of infants without health insurance.

	Percentage	Std. Err.	P-value
United States	17.4		
Rural	19.9	0.003	
Urban	16.8	0.001	0.06
States with Medicaid Expansion	13.4	0.001	
States without Medicaid Expansion	23.4	0.002	0.00
White Infant Households	16.2	0.001	
Black Infant Households	17.4	0.003	
Asian Infant Households	11.4	0.003	
Native American Infant Households	29.5	0.009	
Other/Mixed Infant Households	28.8	0.004	0.00
Hispanic (White Infant Households, Ref.)	34	0.002	0.00

Whether or not Medicaid expanded in the state of household produced significantly (*p* < .01) different levels of infant households without health insurance, in which approximately 13% of infants were in households without insurance in the Medicaid expansion states and approximately 23% were in households without insurance in non-expansion states. Comparing infant households without insurance by race/ethnicity, we found that only 11.4% of Asian, 16.2% of White, and 17.4% of Black infant households reported not having health insurance. These racial groups were much lower than the percentage of Native Americans (29.5%) and Other/Mixed (28.8%) infant households without insurance. However, Hispanic infant household reported the highest percentage of infants born into households without health insurance (over one-third, 34%). Finally, the percentage of Hispanic infant households without health insurance is significantly higher when compared to the percentage of White infant households without health insurance (*p* < .001).

While these statistics provide a general understanding of infant household health insurance, they are merely descriptive, and thus, a multivariate analysis was needed to obtain a complete view of how these variables interact.

Table 2 provides results from the multivariate linear regression and the predicted probability that an infant will be born into a household without any form of health insurance; the way in which it varied by place of residence (i.e., rural or urban and state of residence); and demographic characteristics (e.g., race, marital status, education level, etc.).

**Table 2:** Logistic regression predicting the probability of no health insurance.

	Model 1		Model 2	
**Variable**	**Odds Ratio**	**95% Conf. Interval**	**Odds Ratio**	**95% Conf. Interval**
Rural (Urban Ref.)	1.38*	1.05–1.79	1.13	0.85–1.49
Lives in State without Medicaid Expansion	2.09***	1.82–2.41	1.93***	1.69–2.26
Race/Ethnicity (Non-Hispanic Whites Infant Households Ref.)				
Hispanic Infant Households	1.79***	1.65–1.96	2.13***	1.95–2.34
Black Infant Households	1.07*	1.00–1.14	1.08	0.99–1.15
Asian Infant Households	0.68***	0.63–0.72	0.66***	0.62–0.70
Native American Infant Households	1.86***	1.68–2.05	1.87***	1.70–2.07
Other Infant Households	0.95	0.89–1.01	0.99	0.92–1.06
Marital Status (Married Ref.)				
Separated, Divorced, or Widow	1.80***	1.65–1.96	1.80***	1.58–2.06
Never Married	1.41***	1.37–1.45	1.41***	1.38–1.45
Age	1.00	1.00–1.01	1.00	1.00–1.01
Number of Children	1.00	0.94–1.04	0.99	0.94–1.05
Number of Adults in Household	1.01	0.39–0.99	1.01	0.99–1.03
Education				
Some College or More (High School or Less Ref.)	0.60***	0.47–0.75	0.59***	0.47–0.75
Poor English (Speaks English Well or Better Ref.)	1.93***	1.76–2.11	1.96***	1.80–2.13
Not a U.S Citizen	2.98***	2.39–3.73	2.99***	2.38–3.76
Mother Employed	0.93***	0.91–0.95	0.93***	0.91–0.94
Total Household Income (in 1K $USD)	0.97***	0.97–0.97	0.97***	0.97–0.97
Lives in State without Medicaid Expansion X Rural			1.47***	1.40–1.55
Lives in State without Medicaid Expansion X Hispanic			0.71***	0.67–0.79

After controlling for other factors, the likelihood of being born into a household without health insurance was significantly higher for rural infants than their urban counterparts (OR = 1.38). Similarly, the likelihood that an infant would be born into a household without health insurance in states without the Medicaid expansion was approximately two times greater than that of infants in states with the Medicaid expansion (OR = 2.09). Both of these findings were significant. With respect to race/ethnicity, Model 1 shows that, compared to Non-Hispanic White infant households, the probability of being born into a household without health insurance is higher for Hispanic infant households (OR = 1.79 and *p* < .001), Native American infant households (OR = 1.86 and *p* < .001), and significantly lower for Asians infant households (OR = 0.68 and *p* < .001), but not significantly different for Other/Mixed race infants. Interestingly, Black and Other/Mixed race infants born into households without health insurance were not significantly different when compared to Non-Hispanic White infant households. Marital status, specifically if the mother was separated, divorced, widowed, or had never married, significantly increased the likelihood of an infant being born into a household without health insurance. Lower levels of education, poor English skills, and not being a U.S. citizen also significantly increased the likelihood of an infant household not having health insurance, while maternal employment decreased the likelihood of an infant household not having health insurance.

**Fig 2:**
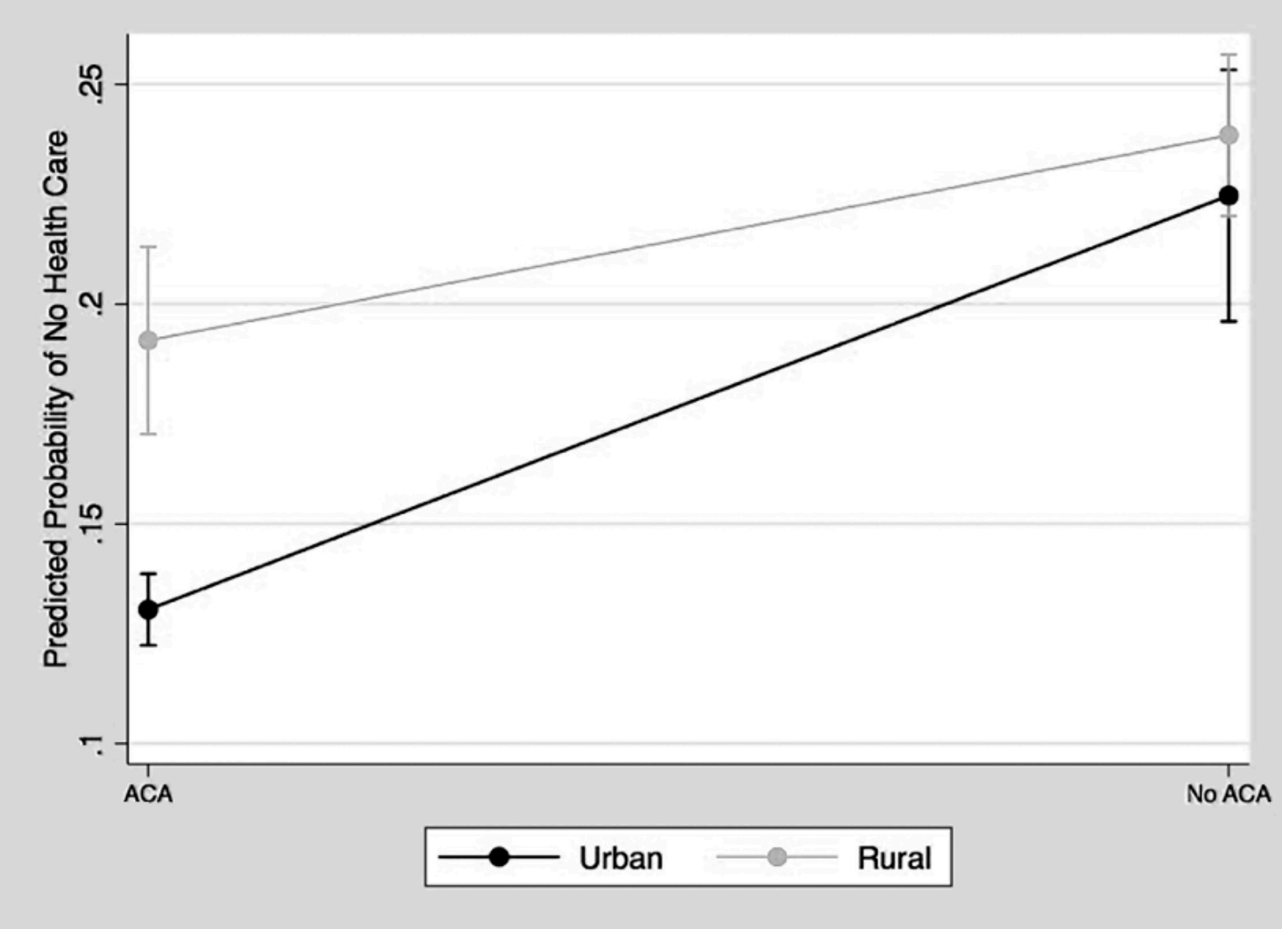
Predicted probability of no health insurance, living in a state without ACA expansion by rurality.

**Fig 3:**
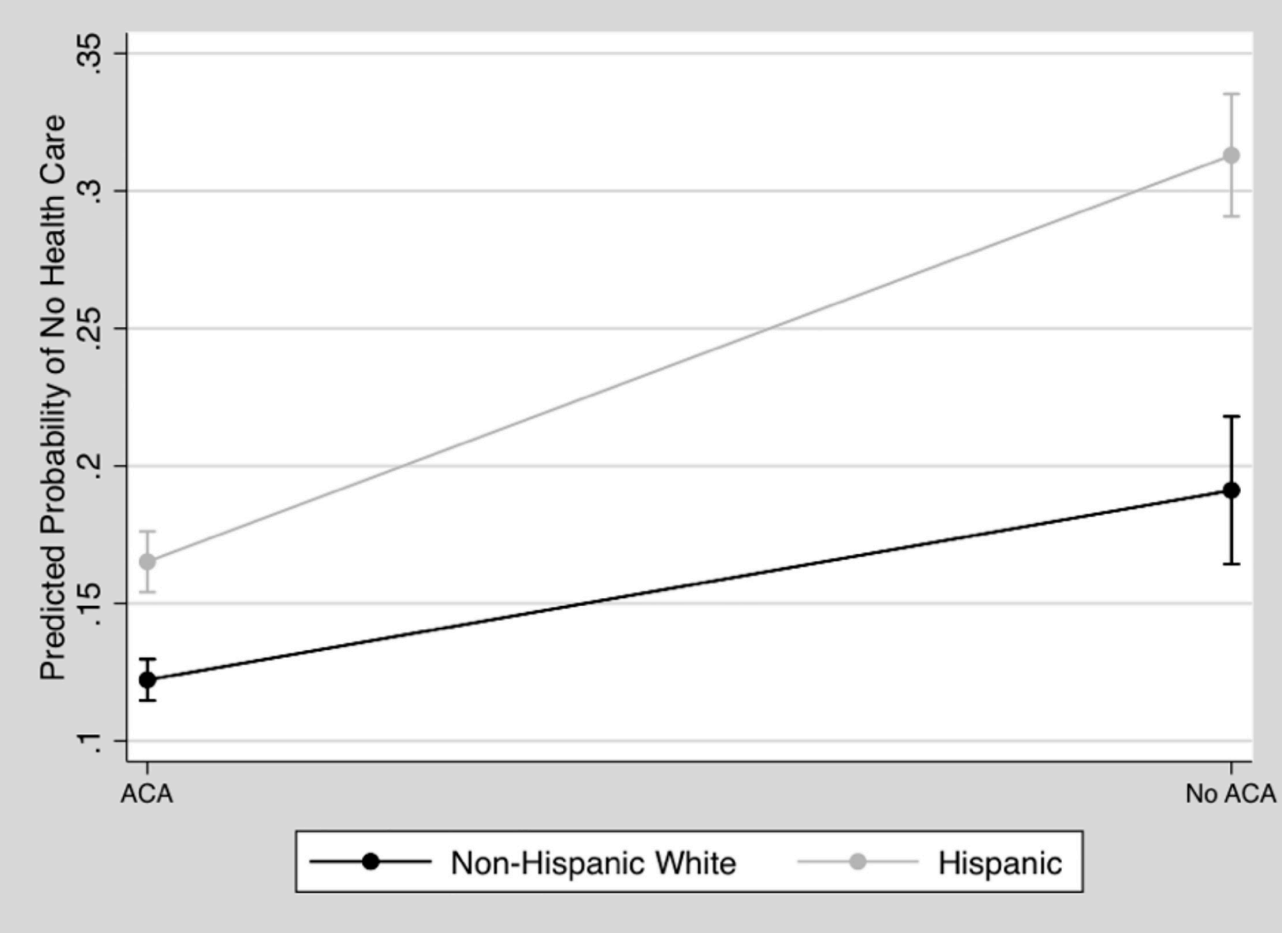
Predicted probability of no health insurance, living in a state without ACA expansion by hispanic about here.

Next, Model 2 included interactions between the variables of rural/urban and the Medicaid expansion, as well as between the variables of rural/urban and Hispanic infant households. We interacted the rural/urban variable with the Medicaid expansion variable for further investigation because both were significant in Model 1, and also because previous research on ACA Medicaid expansion has shown that health insurance coverage varies widely between rural and urban areas [28]. Therefore, we wanted to understand whether or not the Medicaid expansion had similar effects on rural and urban infant households. We also examined the interactions between the Hispanic infant household variables and the Medicaid expansion variable to understand whether or not the expansion of the ACA significantly impacted the effects of race/ethnicity on the probability of an infant being born into a household without health insurance. Further, the U.S. Hispanic population is the fastest-growing ethnic minority group in the United States, but it consistently reports low levels of health insurance coverage [29]. Therefore, having a more detailed understanding of how the Medicaid expansion impacted Hispanic infants’ households provides many important insights for public health and policy practitioners. Figs 2 and 3 present the results of both of these significant interactions from Model 2.

The results in Fig 2 show that the differences between infants born into rural and urban households without health insurance were significant and greater in states with the Medicaid expansion. In states without the Medicaid expansion, the predicted percentage of infants born in rural households without health insurance was only slightly, and so therefore not significantly, higher than that of infants born in urban households without health insurance. Although there is a wider rural/urban gap in states that implemented the Medicaid expansion, both rural and urban infants in Medicaid expansion states were less likely to be born without health insurance than were urban infants in states without the Medicaid expansion.

Fig 3 displays the disparity between Hispanic and Non-Hispanic White infant households that indicated that across Medicaid expansion and non-expansion states, Hispanic infants were significantly more likely than Non-Hispanic White infants to be born into households without health insurance. However, the gap between Hispanic and Non-Hispanic White infant households was narrower in Medicaid expansion states. Also, Hispanic infant households in Medicaid expansion states had a lower predicted probability of not having health insurance than did Non-Hispanic Whites infant households in states without the Medicaid expansion.

## Discussion

This research contributes to the growing body of literature that focuses on recent changes in U.S. health insurance policies, as well as research that has examined the differences in the percentage of rural/urban households without health insurance. The study’s preliminary exploration of the Medicaid expansion focused on the household context of infants aged one year or less, rather than on older children and adolescents. Infants’ health insurance status is often overlooked primarily because of the lack of data. However, by using the fertility question in the American Community Survey (ACS), which is the U.S. Census Bureau’s survey that replaced the decennial census as the primary source for gathering American demographic and household data, our research revealed new racial and spatial inequalities in health insurance that can guide ongoing research and policy. Also, the ACS dataset is sufficiently large for examining trends throughout the United States. Therefore, this research provides valuable insights into the household environments of infants, as well as how their experiences differ based on geography and each mother’s ethnic/racial characteristics.

This study found that there were significant differences in the percentages of having health insurance in infant households between rural/urban areas and ethnic/racial groups. Our findings for infant household health insurance were consistent with previous research that examined the effects of the Medicaid expansion on health insurance coverage overall. However, Asian households with infants were more likely to have health insurance than Non-Hispanic White infant households, a finding that previous research supports [30]. There were also no significant effects for Black infant households, perhaps because there were higher concentrations of Black households in urban areas, which were found to have lower overall percentages of infants without health insurance.

Another critical finding was regarding the higher levels of Hispanic infant households without health insurance. Previous research had shed light on why Hispanic households tend to have lower percentages of health insurance coverage compared to other ethnic/racial groups, even when they were eligible and when it was free or subsidized [31–35]. These findings suggested that there are still additional and perceived barriers to obtaining health insurance among Hispanic populations. Even when households qualify for insurance, these barriers discourage some Hispanic households from applying for it. The significance of the educational, language, and citizenship variables in the logistic regressions suggested that barriers to health insurance persist across and beyond the ethnic identification of households with infants.

Despite the continued prevalence of these obstacles, the Medicaid expansion succeeded in reducing the percentage of uninsured Hispanic infants. In states that did not expand Medicaid, over one-third of Hispanic infants were born into households without health insurance, while in states that did, this percentage was reduced by nearly a half. Together with improving the percentage of Hispanic households overall that report having health insurance coverage, the Medicaid expansion also significantly reduced the health insurance disparity between Hispanic infant households and Non-Hispanic White infant households. In a country that is still working to overcome ethnic bias in many areas, it is essential to consider the Medicaid expansion as an effective way to reduce health-related inequalities for Hispanic infants.

Although the Medicaid expansion bridged the racial gap in terms of the percentage of households with health insurance, the effect on the rural/urban gap was mixed. While expansion decreased the percentage of infants without health insurance overall in both rural and urban areas, it did so by a much smaller margin for rural infants. Despite resources from the ACA to address the needs of rural communities, rural/urban disparities in the percentages of households without health insurance persisted [13]. Differences in the percentage of rural/urban households with insurance have been previously attributed to the fact that fewer rural than urban states had expanded Medicaid [12]. However, our research showed that the disparity remains even in states that did expand. Continued differences in the percentage of rural/urban households without health insurance in Medicaid expansion states suggests that continued provisions and efforts are needed to reduce rural/urban disparities in healthcare access.

A limitation of our study is the lack of data on the different kinds of health insurance coverage. Although we know which states expanded their Medicaid coverage, this research cannot isolate the insurance expansion that occurred through the ACA insurance exchange. Additionally, the 2011–2015 ACS data overlaps the Medicaid expansion. As a result, this research cannot completely isolate the effects of the Medicaid expansion. Due to this limitation, we caution readers when interpreting our results. However, since Medicaid expansion is an important and ongoing issue, we believe are preliminary exploratory analysis can provide useful insights that can inform the current debate over Medicaid expansion.

Additional research is needed in order to understand the changes in the kind of health insurance more fully. Currently available ACS data is not able to track the conditions that these children face in early childhood and beyond. As a result, this research cannot evaluate the developmental consequences of being born into a household without health insurance. However, a growing number of studies show that a lack of health care access can severely impact children [4–6]. Furthermore, although we cannot define the specific development consequences of being born without health insurance, our findings do help to identify how health care access varies demographically and geographically. These findings can be valuable to families and policymakers in states that continue to debate Medicaid expansion.

Understanding the long-term health and developmental costs of infants born into households without health insurance is a crucial issue that future research needs to address. Furthermore, our research examined infants at the state level in order to evaluate the Medicaid expansion’s effect on health insurance. However, the environmental effects on child development occur at multiple and often overlapping levels, ranging from the neighborhood to the community to the state [36]. Future research and micro-data examination of the well-being of infants is needed in order to understand the long-term impact of the Medicaid expansion more fully.

## Conclusion

Understanding infant health insurance across the rural/urban spectrum, as well as ethnic/racial groups, will become increasingly important as the U.S. population becomes increasingly urban and transitions to a minority-majority. Although lacking health insurance does not mean that infants cannot receive healthcare from providers such as free public clinics, infants born to mothers without health insurance face significant barriers in terms of accessing health care. By far from a perfect solution, our findings showed that, overall, the Medicaid expansion benefited infants across the rural/urban spectrum and ethnic/racial groups. Although thousands of infants were disadvantaged by being born in states without the Medicaid expansion, the continued increase in health insurance coverage through the Medicaid expansion passed by state legislators and referendums was a positive step that helped to ensure that more infants are not born disadvantaged because they lacked health insurance. Although this research examined only the first two years of the Medicaid expansion under the ACA, our findings provide policymakers with a baseline and many valuable insights into how future changes to the ACA and the Medicaid expansion can impact households and infants.

## References

[1] Assaf S, Campostrini S, Gotway Crawford C, Di Novi C, Xu F. Analyzing Disparities Trends for Health Care Insurance Coverage Among Non-Elderly Adults in the US: Evidence from the Behavioral Risk Factor Surveillance System, 1993–2009. The European Journal of Health Economics. 2014;18 3: 387–398. 10.1007/s10198-016-0806-1 PMC10884940 27241187

[2] Sommers BD, Gunja MZ, Finegold K, Musco T. Changes in self-reported insurance coverage, access to care, and health under the Affordable Care Act. Jama. 2015;314 4: 366–374. 10.1001/jama.2015.8421 26219054

[3] Blumenthal D, Collins SR. Health care coverage under the Affordable Care Act—a progress report. The New England Journal of Medicine. 2014:371 3: 275–281. 10.1056/NEJMhpr1405667 24988300

[4] Manatt P, Phillips LLP. Medicaid's Impact on Health Care Access, Outcomes and State Economies. Robert Wood Johnson Foundation. 2019.

[5] Clotfelter CT, Ladd HF, Vigdor JL. New destinations, new trajectories? The educational progress of Hispanic youth in North Carolina. Child development. 2012;83 5: 1608–1622. 10.1111/j.1467-8624.2012.01797.x 22966926

[6] Duncan GJ, Ziol‐Guest KM, Kalil A. Early‐childhood poverty and adult attainment, behavior, and health. Child development. 2010;8 1: 306–325. 10.1111/j.1467-8624.2009.01396.x 20331669

[7] Borjas GJ. Poverty and program participation among immigrant children. The Future of Children. 2011;21 1: 247–266. 10.1353/foc.2011.0006 21465863

[8] Call KT, McAlpine DD, Garcia CM, Shippee N, Beebe T, Adeniyi TC, et al Barriers to care in an ethnically diverse publicly insured population: is health care reform enough? Medical care. 2014;52 8: 720–727. 10.1097/MLR.0000000000000172 25023917

[9] Curtin SC, Osterman MJ, Uddin SF, Sutton SR, Reed PR. Source of payment for the delivery: births in a 33-state and District of Columbia reporting area. 2010. National Vital Statistics Reports: From the Centers for Disease Control and Prevention, National Center for Health Statistics. National Vital Statistics System. 2013;62 5: 1–20. 24364892

[10] Ziller EC, Coburn AF, Anderson NJ, Loux SL. Uninsured rural families. The Journal of Rural Health. 2008;24 1: 1–11. 10.1111/j.1748-0361.2008.00131.x 18257865

[11] Ziller EC., Lenardson, JD, Coburn AF. Rural adults delay, forego, and strategize to afford their pre-ACA health care. 2015.

[12] Newkirk V, Damico A. The Affordable Care Act and insurance coverage in rural areas. The Kaiser Commission on Medicaid and the Uninsured. 2014.

[13] Benitez JA., Seiber EE. US Health Care Reform and Rural America: Results From the ACA's Medicaid Expansions. The Journal of Rural Health. 2018;34 2: 213–222. 10.1111/jrh.12284 29105809

[14] Kwon D. Should you take an app for that. Scientific American. 2015.

[15] Karpman M, Zuckerman S, Kenney GM, Odu Y. QuickTake: Substantial Gains in Health Insurance Coverage Occurring for Adults in Both Rural and Urban Areas. 2015.

[16] Hargraves JL, Hadley J. The contribution of insurance coverage and community resources to reducing racial/ethnic disparities in access to care. Health services research. 2003;38 3: 809–829. 10.1111/1475-6773.00148 12822914 PMC1360918

[17] Lillie-Blanton M, Hoffman C. The role of health insurance coverage in reducing racial/ethnic disparities in health care. Health affairs. 2005;24 2; 398–408. 10.1377/hlthaff.24.2.398 15757923

[18] Buchmueller TC, Levinson ZM, Levy HG, Wolfe BL. (2016). Effect of the Affordable Care Act on racial and ethnic disparities in health insurance coverage. American journal of public health. 2016;106 8: 1416–1421. 10.2105/AJPH.2016.303155 27196653 PMC4940635

[19] Monnat SM. Hispanic Health Insurance Rates Differ between Established and New Hispanic Destinations. 2016. https://w3001.apl.wisc.edu/pdfs/b02_16.pdf

[20] Ruggles S, Flood S, Goeken R, Grover J, Meyer E, Pacas J, et al IPUMS USA: Version 8.0 [dataset]. Minneapolis, MN: IPUMS, 2018 10.18128/D010.V8.0

[21] Lichter DT., Sanders SR, Johnson KM. Hispanics at the starting line: poverty among newborn infants in established gateways and new destinations. Social Forces. 2015;94 1: 209–235.

[22] Bliss J, Jensen N, Thiede B, Shoham J, Dolan C, Sibson V, et al Factors associated with the risk of acute malnutrition among children aged 6 to 36 months in households targeted by an emergency cash transfer program. Food and nutrition bulletin. 2016;37 3: 387–400. 10.1177/0379572116654772 27402641

[23] Garrett AB, Gangopadhyaya A. Who gained health insurance coverage under the ACA, and where do they live?. Urban Institute, ACA Implementation—Monitoring and Tracking. 2016. [Map 1].

[24] Young IM. Gender as seriality: Thinking about women as a social collective. Gender and Justice, Routledge. 2017; 3–28.

[25] Hoffmann JP. Regression models for categorical, count, and related variables: An applied approach. Oakland: Univ of California Press; 2016.

[26] Mitchell MN. Interpreting and visualizing regression models using Stata (No. 005.369 M58.). College Station, TX: Stata Press; 2012.

[27] Lichter DT, Johnson KM, Turner RN, Churilla A. Hispanic Assimilation and Fertility in New US Destinations. International Migration Review. 2012;46 4: 767–791. 10.1111/imre.12000 23325987 PMC3544406

[28] Soni A, Hendryx M, Simon K. Medicaid expansion under the Affordable Care Act and insurance coverage in rural and urban areas. The Journal of Rural Health. 2017;33 2: 217–226. 10.1111/jrh.12234 28114726

[29] Monnat SM. The new destination disadvantage: disparities in Hispanic health insurance coverage rates in metropolitan and nonmetropolitan new and established destinations. Rural sociology. 2017;82 1: 3–43. 10.1111/ruso.12116 28479612 PMC5415350

[30] Bloom B, Black LI. Health of Non-Hispanic Asian Adults: United States, 2010–2014. NCHS data brief 2016;247: 1–8. 27227734

[31] Clemans-Cope L, Kenney GM., Buettgens M, Carroll C, Blavin F. The Affordable Care Act’s coverage expansions will reduce differences in uninsurance rates by race and ethnicity. Health Affairs. 2012;31 5: 920–930. 10.1377/hlthaff.2011.1086 22566430

[32] Currie J. The Take Up of Social Benefits. [Working Paper No. 10488] National Bureau of Economic Research, Cambridge, MA 2004.

[33] Derose KP, Escarce JJ, Lurie N. Immigrants and health care: sources of vulnerability. Health Affairs. 2007;26 5: 1258–1268. 10.1377/hlthaff.26.5.1258 17848435

[34] Gee ER. Eligible Uninsured Latinos: 8 in 10 Could Receive Health Insurance Marketplace Tax Credits, Medicaid, or CHIP. Washington DC: Department of Health and Human Services, Office of Assistant Secretary for Planning and Evaluation. 2014.

[35] Ku L, Matani S. Left out: immigrants’ access to health care and insurance. Health Affairs. 2001;20 1: 247–256. 10.1377/hlthaff.20.1.247 11194848

[36] Thiede BC, Sanders SR, Lichter DT. Born Poor? Racial Diversity, Inequality, and the American Pipeline. Sociology of Race and Ethnicity. 2018;4 2: 206–228.

